# Evaluation of high mobility group box 1 protein as a presurgical diagnostic marker reflecting the severity of acute appendicitis

**DOI:** 10.1186/1757-7241-20-61

**Published:** 2012-09-04

**Authors:** Chuanxin Wu, Hang Sun, Hongliang Wang, Junmeng Chi, Qi Liu, Hui Guo, Jianping Gong

**Affiliations:** 1 Department of Hepatobiliary Surgery, The Second Affiliated Hospital of Chongqing Medical University, Chongqing, 400010, China; 2 Key Laboratory of Molecular Biology for Infectious Diseases, Ministry of Education, Liver Diseases Research and Treatment Center the Second Affiliated Hospital of Chongqing Medical University, Chongqing, 400010, China

**Keywords:** High mobility group box 1, High-sensitivity C-reactive protein, Acute appendicitis

## Abstract

**Objectives:**

To validate the role of high mobility group box-1(HMGB1) in diagnosis of acute appendicitis (AA) with different pathological severity.

**Methods:**

According to the pathologically diagnosis, 150 patients underwent appendectomies between Jan. 2007 and Dec, 2010 were divided into acute simple, acute suppurative and acute gangrenous appendicitis as group 1, 2 and 3, respectively. Each patient group contains 50 sex and age matched cases to make comparison with 50 healthy volunteers. The mRNA and protein expression levels of serum HMGB1 were determined by real-time quantitative PCR and enzyme linked immunosorbent assay (ELISA). Serum High-sensitivity C-reactive protein (hs-CRP) levels were determined by rate nephelometric immunoassay.

**Results:**

In comparison with health volunteers, relative HMGB1 mRNA levels in group 1, 2 and 3 were significantly increased 3.05 ± 0.51,8.33 ± 0.75 and 13.74 ± 1.09 folds, reflecting a tendency of augmented severity. In accordance, serum protein levels of HMGB1 were 10.97 ± 1.64, 14.42 ± 1.56 and 18.08 ± 2.41 ng/ml in 3 patient groups, which are significantly higher than that of healthy volunteers’ 5.47 ± 0.73 ng/ml. hs-CRP levels were 12.85 ± 3.41, 21.04 ± 1.98 and 31.07 ± 5.46 ng/ml in 3 patients groups compared with 2.06 ± 0.77 ng/ml in controls. The concentrations of HMGB1 and hs-CRP were both positively correlated with disease severity.

**Conclusion:**

Serum HMGB1 constitutes as a valuable marker in diagnosis of AA. Positively correlated with hs-CRP level, mRNA and protein expression of HMGB1 to a certain extent reflected the severity of AA.

## Introduction

Acute appendicitis is the most common acute abdominal disease in general surgeries of patients with typical clinical symptoms. The stages of AA can be divided into simple, suppurative, gangrenous, perforated, recurrent, and chronic etc. according to the severity of the diseases. Although AA is not a life-threatening disease, mortality is still reported as high as 1%. Diagnosis of AA is often a clinical challenge because it can mimic several abdominal pain conditions. So far no single symptom or diagnostic test can accurately confirm the diagnosis of AA in all cases. The emergencies are sometimes misdiagnosed or missed. Although the surgery appendectomy itself is a relatively safe surgical procedure, rates of negative appendectomy and perforated appendicitis remain fairly high, around 10–30 percent, with a higher rate of ~40% in high risk populations such as women of reproductive age. For a long time, the preoperative diagnosis of AA is largely relied on the clinical assessment, previous experience, total white blood cell (WBC) counts, neutrophil percentage or a combination of several examinations. These traditional methods are rather nonspecific. Recently ultrasonography and computed tomography were applied in diagnosis of AA. However, it is still controversial if these imaging aided applications have actually resulted in significantly reduced cases of negative appendectomy and perforated appendicitis [1,2]. Therefore, identification of novel diagnostic markers of AA is clinically highly desirable, which can to a certain extent reflect the severity of the AA condition.

HMGB1 is recently identified inflammatory factor which plays a crucial role in the late stage of severe sepsis. It is highly expressed in severe sepsis, acute pancreatitis, hemorrhagic shock, severe trauma and acute lung injury. And its expression is closely correlated with the severity of these symptoms [3-5]. Recent study has demonstrated that HMGB1 level was increased in serum sample of AA patients. Although preliminary, it was indicated that HMGB1 is a potential candidate to aid in AA diagnostic [6].

In this study, we have evaluated the mRNA and protein expression level of HMGB1 in AA patients. In addition, HMGB1 was assessed to be a diagnostic marker for AA patients with differential pathological severity of the disease, aiming to provide a valuable marker to improve the preoperative diagnosis of AA for clinical use.

## Material and methods

### Study population

According to the pathologically diagnosis, 150 patients underwent appendectomies between Jan. 2007 and Dec 2010 were divided into acute simple, acute suppurative and acute gangrenous appendicitis as group 1, 2 and 3, respectively. All experimental protocols were approved by the local research ethics committee of the Second Affiliated Hospital of Chongqing Medical University and complied with the China government guidelines. There are randomized chosen 25 males and 25 females in each AA group. The average age for acute simple, acute suppurative and acute gangrenous appendicitis groups are 37.63 ± 14.68, 34.72 ± 15.45 and 36.63 ± 15.98 respectively. 50 healthy volunteers (25 males and 25 females, average age 38.77 ± 13.29) without any abdominal syndromes were used as a control group. Blood samples of the patients were withdrawn when admitted to the hospital. White blood cells were counted and the blood samples were stored for future use. No significant differences were observed in age, family history and other medical conditions between groups.

### Serum HMGB1

Serum HMGB1 concentrations were measured by ELISA with human HMGB1 detection ELISA kit (SUNBIO (Cat# HE017), Beijing, China) according to the manufacturer’s instruction.

### Relative mRNA expression level

Peripheral blood total RNA was isolated with total RNA isolation kit purchased from Bioteke, Beijing, China, according to manufacturers’ instruction. RNA was reverse transcribed using PrimeScript® RT reagent Kit (Perfect Real Time) from TaKaRa biotech, Dalian, China. SYBR® Premix Ex Taq™ (Perfect Real Time) from TaKaRa was used for quantitative real-time PCR. The primers for human HMGB1 were designed with Primier4.1 software, GADPH was used as a reference gene. Both primers were synthesized by TaKara. Primers used for human HMGB1 are, forward: 5’-GCG GAC AAG GCC CGT TA-3’; reverse: 5’-AGA GGA AGA AGG CCG AAG GAA-3’; Amplicon size 119 bp. Human GADPH primers are Forward: 5’- TGC CAA ATA TGA TGA CAT CAA GAA-3’; Reverse: 5’- GGA GTG GGT GTC GCT GTT G-3’; Amplicon size 121 bp. Relative gene expression was calculated to that of GAPDH on the basis of ΔCt value. Data with 2^-▽▽CT^ > 2, were considered to be statistically significant.

### Hs-CRP concentration

Serum Hs-CRP concentrations were determined locally in clinical laboratory of the hospital with rate nephelometric immunoassay.

### Statistical analysis

Values were expressed as the standard deviations of the mean. Data were analyzed with SPSS 13.0 software. Group means did not exhibited as normal distribution and were examined using Kruskal-Wallis test. The P values of less than 0.05 were considered to be statistically significant.

## Results

### HMGB1 protein concentration in AA patients

**Figure 1:**
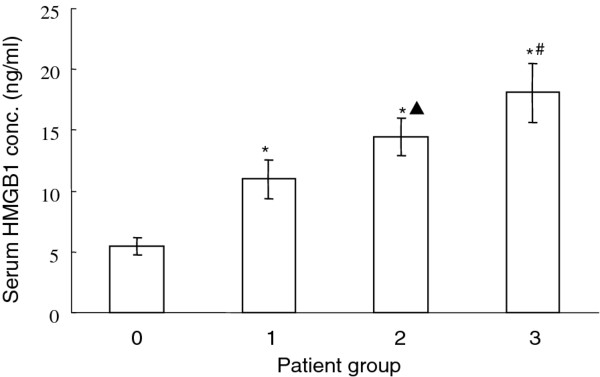
**Serum HMGB1 concentration in patient groups of acute simple (group 1), acute suppurative (group 2) and acute gangrenous appendicitis (group 3) in comparison with healthy volunteers (group 0).** *, P<0.01 compared with healthy volunteers; *▴* P<0.01 compared with acute simple (group 1) patients; # P<0.01 compared with acute suppurative (group 2) patients.

The average HMGB1 protein level in acute simple (group 1), acute suppurative (group 2) and acute gangrenous appendicitis (group 3) are 10.97 ± 1.64 ng/ml, 14.42 ± 1.56 ng/ml and 18.08 ± 2.41 ng/ml,which is significantly higher than that of health volunteer group at 5.47 ± 0.73 ng/ml (*P* <0.01). The HMGB1 concentration in acute simple appendicitis group is almost doubled compared with healthy controls. And acute gangrenous appendicitis is about 4 folds higher in HMGB1 secretion. Importantly, HMGB1 concentration in group 2 is significantly higher than that of group 1, and group 3 is much higher than group 2 (P < 0.01, Figure 1). It is indicated that with the exacerbated inflammation and worsen of the disease, HMGB1 protein level in the peripheral blood is significantly increased and is correlated with the severity of AA.

### HMGB1 mRNA expression in AA patients

**Figure 2:**
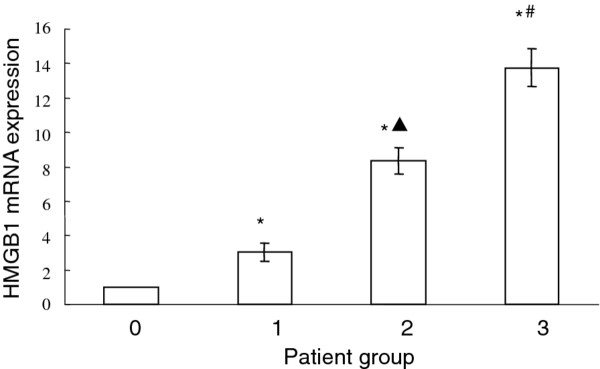
**Serum HMGB1 relative mRNA expression in patient groups of acute simple (group 1), acute suppurative (group 2) and acute gangrenous appendicitis (group 3) in comparison with healthy volunteers (group 0).** *, P<0.01 compared with healthy volunteers; *▴* P<0.01 compared with acute simple (group 1) patients; # P<0.01 compared with acute suppurative (group 2) patients.

In comparison with that of health volunteers, the HMGB1 mRNA expression level of patients in acute simple (group 1), acute suppurative (group 2) and acute gangrenous appendicitis (group 3) are 3.05 ± 0.51,8.33 ± 0.75 and 13.74 ± 1.09 (Figure 2). The 2^-▽▽CT^ value of HMGB1 in all three AA groups are higher than 2,indicating that compared with healthy controls, the HMGB1 expression is significantly increased. In addition, HMGB1 expression in group 2 is significantly higher than that of group1 (P < 0.01), and group 3 is significantly higher than group 2 (P < 0.01), indicating that the increased HMGB1 level is positively correlated with the severity of the AA conditions.

### Serum Hs-CRP concentrations in AA patients

**Figure 3:**
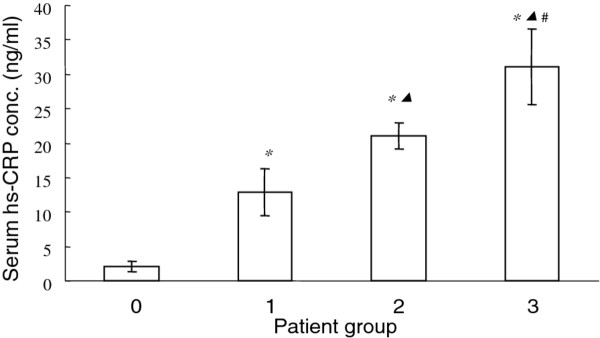
**Serum Hs-CRP concentration in patient groups of acute simple (group 1), acute suppurative (group 2) and acute gangrenous appendicitis (group 3) in comparison with healthy volunteers (group 0).** *, P<0.01 compared with healthy volunteers; *▴* P<0.01 compared with acute simple (group 1) patients; # P<0.01 compared with acute suppurative (group 2) patients.

CRP levels in acute simple (group 1), acute suppurative (group 2) and acute gangrenous appendicitis (group 3) are 12.85 ± 3.41 ng/ml, 21.04 ± 1.98 and 31.07 ± 5.46 ng/ml, which are all significantly higher than that of healthy volunteer group at 2.06 ± 0.77 ng/ml(*P* <0.01). Hs-CRP concentration in group 2 is significantly higher than that of group 1 and group 3 is higher than group 2 (P < 0.01, Figure 3). Hs-CRP level also exhibits a positive correlation with the severity of AA.

### Peripheral blood white blood cell counts in AA patients

**Figure 4:**
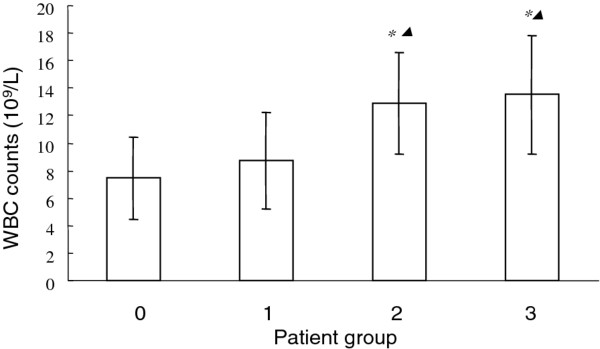
**White blood cell counts of acute simple (group 1), acute suppurative (group 2) and acute gangrenous appendicitis (group 3) in comparison with healthy volunteers (group 0).** *, P<0.01 compared with healthy volunteers; *▴* P<0.01 compared with acute simple (group 1) patients.

White blood cells counts of acute suppurative (group 2) and acute gangrenous appendicitis (group 3) are 12.90 ± 3.69 × 10^9^ and 13.53 ± 4.31 × 10^9^, respectively, which are both significantly higher than that of acute simple appendicitis patients (group 1) at 8.73 ± 3.49 × 10^9^ and healthy volunteer group at 7.45 ± 2.98 × 10^9^(*P* <0.01). Importantly, we did not observe a significant increase in simple appendicitis patients group and healthy volunteer group (*P* >0.05), demonstrating that WBC is not a bona fide diagnostic marker for AA, especially at the early stage of AA progression. We also did not observe a significant difference in WBC counts between group 1 and 2, indicating that WBC counts are not very sensitive to reflect the severity of AA (Figure 4).

### Correlation analysis between serum HMGB1 and Hs-CRP in AA patients

**Figure 5:**
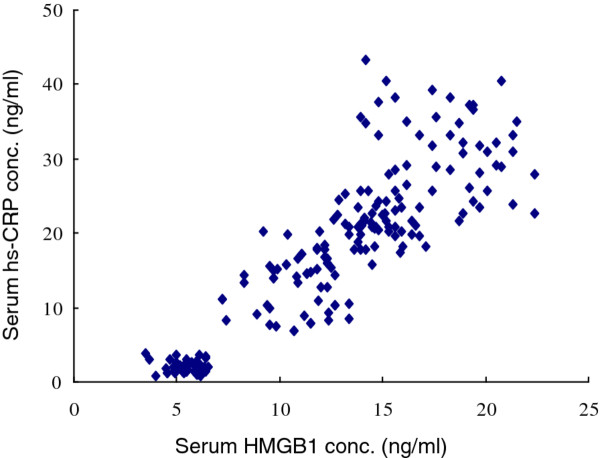
**Correlation analysis between serum HMGB1 and Hs-CRP in AA patients.** r=0.901, P <0.01.

Statistical analysis demonstrated that in AA patients, serum HMGB1 and Hs-CRP are positively correlated. Both factors are increased with the development of AA and in accordance with the severity of the disease (r = 0.901, P <0.01, Figure 5).

## Discussion

HMGB1 is a very abundant chromatin-binding protein residing in the eukaryotic cell nucleus. Originally identified as a chromosomal protein regulating gene transcription, HMGB1 is one of the most evolutionary conservative proteins in the eukaryotic organism. It functions to stabilize nucleosome formation, to promote the binding of transcription factors to target DNA sequence, and to play an important role in DNA recombination, DNA repair and gene transcriptional activation [7]. Inside nucleus, HMGB1 is a potent DNA binding factors but outside of the cells it acts as an inflammatory mediator. HMGB1 is shown to be actively secreted by activated monocytes or macrophages. In addition, it can also be passively released from necrotic or damaged cells to act as a potent cytokine to trigger inflammation. The secreted or leaked HMGB1 in the outside of the cellular membrane, as an important inflammatory mediator in late stage sepsis, is highly expressed in various inflammatory diseases and in severe sepsis. In addition, HMGB1 was considered to play a crucial role in endotoxin induced death effects in patients [8,9].

AA is characterized by acute suppurative infection. When left untreated, AA can cause systemic inflammatory symptoms and has the potential to develop into severe complications such as perforation or sepsis. Although AA is not a disease with high mortality, diagnosis of AA is often misdiagnosed or missed. Unnecessary appendectomy can cause surgical risks for patients and waste of the money for the societies. Currently available preoperative diagnosis of AA is either auxiliary or nonconfirmative. Identification of novel diagnostic markers of AA is clinically highly desired. Unfortunately not much attention had been paid to connect HMGB1 and AA preoperative diagnosis.

In this study, we have investigated the diagnostic potential of HMGB1, both at mRNA and protein levels in AA patients. The average HMGB1 mRNA and protein levels in acute simple, acute suppurative and acute gangrenous appendicitis group are significantly higher than that of health volunteer group. Importantly, HMGB1 concentration in acute suppurative group is significantly higher than that of acute simple appendicitis. Similarly, acute gangrenous appendicitis group is much higher than the other 2 less severe AA. It is indicated that with the exacerbation of AA, HMGB1 mRNA and protein levels are positively correlated with the severity of the disease.

We have also examined the PBMC WBC counts in patients with various pathological severities in comparison with health volunteers. It is showed that although the WBC counts in patients groups of acute suppurative and acute gangrenous appendicitis are significantly higher than that in acute simple of healthy volunteers, there is no significant difference between these 2 patient groups. Importantly, there is barely any significant difference in WBC counts between acute simple appendicitis patients and healthy volunteers, indicating that using this index can lead to missed treatment and rather not sensitive. Clearly WBC can not reflect the severity of the AA condition. In comparison, the mRNA and protein level of HMGB1 in acute simple patients are significantly higher than healthy volunteers. It suggests that detection of serum HMGB1 level is instrumental and confirmative assay to diagnose AA. It will be especially valuable to diagnose patients with AA syndrome but with normal WBC counts.

After infection, both bacteria produced endotoxins and various cytokines released by activated monocytes/macrophages such as TNFα, IL1β, IFNγ etc. can promote HMGB1 translocation from nucleus to cytoplasmic organelles. Eventually HBGB1 is secreted or released to the extracellular area and further promote the transcriptional synthesis of HMGB1 itself to provide additional backup [10-12]. In addition, released HMGB1 itself acts as a potent cytokine which can stimulate multiple proinflammatory cytokine release and can worsen the inflammation. From this point of view, HMGB1 level to a certain extent should be able to reflect the severity of sepsis and infectious disease such as AA, useful to be a diagnostic marker. It has been shown that inhibition the expression and release of HMGB1 can attenuate the organ injury in multiple diseases, especially in severe sepsis [9,13]. In acute AA, bacteria invade and infect the wall of the appendix, causing releasing of bacterial endotoxin and proinflammatory cytokines. These factors can potently activate monocytes/macrophages to release HMGB1, followed by an elevated PBMC HMGB1 expression and secretion. The released HMGB1 can not only worsen the local inflammation but also affect the distal organ damage. Therefore, HMGB1 might play an important role in the pathological progress of AA, and can be clinically treated as a therapeutic target. Controlling its expression and release might improve the AA therapy efficacy, manage systemic inflammation and reduce the organ injury from severe sepsis.

Several recently studies have tried to identify sensitive diagnostic markers reflecting the severity of AA, such as bilirubin and procalcitonin levels. In a prospective study, Procalcitonin, an established marker of inflection and sepsis, only increased with rare case of AA [14]. It is remarkably low sensitivity prevented its clinical use as diagnostic marker of AA. A recent study by Wu et al. confirmed that procalcitonin test might not be useful for AA patients [15]. Another promising marker is bilirubin. Hyperbilirubinemia was assessed as a predictive marker for appendiceal perforation in a large group of 538 patients, showing very high specificity and reasonably good sensitivity [16]. However, another study has demonstrated that bilirubin is inferior to CRP for anticipation of perforation of AA [17]. Therefore, novel markers that can predict the severity of AA are still highly desirable. Hs-CRP concentration increases in various injury and inflammatory diseases, such as cardiovascular diseases. It is deemed to be a risk factor for patients with heart disease. Its serum concentration to a certain extent reflects the severity of the inflammation; however, it is rather nonspecific as it cannot pinpoint the location of the inflammation. In addition, it is also an indicator of system inflammation. Therefore, combination detection with other markers to diagnose AA is necessary, especially for those patients who have cardiovascular diseases. In our study, Hs-CRP levels in acute simple, acute suppurative and acute gangrenous appendicitis patients are all significantly higher than that of healthy volunteers. Hs-CRP concentration in acute suppurative is significantly higher than that of acute simple, and Hs-CRP of acute gangrenous appendicitis patients is remarkably higher than acute suppurative group (P < 0.01, Figure 3). Hs-CRP level exhibits a positive correlation with the severity of AA. As serum HMGB1 and Hs-CRP are positively correlated with the severity of AA, a combination use of both markers will be useful for the evaluation and conformation of the severity of the diseases before operation.

In conclusion, as HMGB1 plays a crucial role in the pathophysiological process of inflammatory diseases such as AA, serum HMGB1 level constitutes a novel marker in the diagnosis of AA. It even holds promise to help doctors to judge the severity of the acute syndrome under different stage of AA with A combination detection of HMGB1 and Hs-CRP.

## Competing interests

The author(s) declare that they have no competing interests.

## Authors’ contributions

WC and SH designed and performed the study, acquired and analyzed the data, and drafted the manuscript. WH and CJ selected the patients and collected specimens. GH carried out the immunoassays. GJ and LQ designed the study and revised the manuscript critically. All authors read and approved the final manuscript.

## Sources of funding

This study was financially supported by grants from the National Natural Science Foundation of China (no.81071339, 81171543) and the Traditional Chinese Medicine Science and Technology Research Project of Chongqing Health Bureau (2009-2-148).
